# Ocean Acidification-Induced Food Quality Deterioration Constrains Trophic Transfer

**DOI:** 10.1371/journal.pone.0034737

**Published:** 2012-04-11

**Authors:** Dennis Rossoll, Rafael Bermúdez, Helena Hauss, Kai G. Schulz, Ulf Riebesell, Ulrich Sommer, Monika Winder

**Affiliations:** 1 Helmholtz Centre for Ocean Research Kiel (GEOMAR), Kiel, Germany; 2 Department of Systems Ecology, Stockholm University, Stockholm, Sweden; National Institute of Water & Atmospheric Research, New Zealand

## Abstract

Our present understanding of ocean acidification (OA) impacts on marine organisms caused by rapidly rising atmospheric carbon dioxide (CO_2_) concentration is almost entirely limited to single species responses. OA consequences for food web interactions are, however, still unknown. Indirect OA effects can be expected for consumers by changing the nutritional quality of their prey. We used a laboratory experiment to test potential OA effects on algal fatty acid (FA) composition and resulting copepod growth. We show that elevated CO_2_ significantly changed the FA concentration and composition of the diatom *Thalassiosira pseudonana*, which constrained growth and reproduction of the copepod *Acartia tonsa*. A significant decline in both total FAs (28.1 to 17.4 fg cell^−1^) and the ratio of long-chain polyunsaturated to saturated fatty acids (PUFA:SFA) of food algae cultured under elevated (750 µatm) compared to present day (380 µatm) *p*CO_2_ was directly translated to copepods. The proportion of total essential FAs declined almost tenfold in copepods and the contribution of saturated fatty acids (SFAs) tripled at high CO_2_. This rapid and reversible CO_2_-dependent shift in FA concentration and composition caused a decrease in both copepod somatic growth and egg production from 34 to 5 eggs female^−1^ day^−1^. Because the diatom-copepod link supports some of the most productive ecosystems in the world, our study demonstrates that OA can have far-reaching consequences for ocean food webs by changing the nutritional quality of essential macromolecules in primary producers that cascade up the food web.

## Introduction

Anthropogenic emissions of carbon dioxide (CO_2_) and its uptake by the surface ocean cause profound changes in marine carbonate chemistry, including seawater acidification and lowering of the calcium carbonate saturation state [1], [2]. Contemporary surface ocean pH has decreased on average by 0.1 units due to CO_2_ invasion since preindustrial times. According to IPCC projections atmospheric partial pressure of CO_2_ (*p*CO_2_) is expected to further increase from current ∼390 µatm to ∼760 µatm, corresponding to a drop in mean oceanic surface pH by 0.3 to 0.4 units until the end of the 21^st^ century (‘business-as-usual scenario’ [3], [4]). This change in carbonate chemistry, termed ocean acidification (OA), is thought to primarily affect calcifying organisms building their shells and skeletons of calcium carbonate [5], [6], [7]. Biological effects of OA on non-calcifying organisms are diverse and often highly species-specific [8].

Our present understanding of potential OA impacts is almost entirely limited to single species responses, while OA consequences for food web interactions remain poorly understood. Indirect impacts through trophic interactions are expected because OA may change the biochemical composition of primary producers that affects nutritional food quality for consumers. Increased CO_2_ can stimulate carbon fixation by photosynthetic organisms and thereby reduce the nutrient content relative to carbon [9], [10], [11], which determines the food quality for herbivores [12]. Enhanced carbon consumption relative to nutrients under elevated CO_2_ conditions [13], [14] can cause an imbalance between phytoplankton stoichiometric composition and consumer nutrient demand for somatic growth [11]. Besides elemental stoichiometry, fatty acid (FA) associated food quality is a critical factor that regulates the energy transfer between primary producers and consumers [15], [16], because essential FAs cannot be synthesized *de novo* by heterotrophic organisms and have to be acquired through the diet. In particular long-chain polyunsaturated FAs (PUFAs) such as docosahexaenoic acid (DHA), eicosapentaenoic acid (EPA) and arachidonic acid (ARA) play an important role in growth, development and reproduction success in heterotrophs [17], [15]. OA may impact phytoplankton FA synthesis because extracellular pH is known to affect various intracellular physiological parameters [18] that influences enzyme activity.

The classic diatom-copepod-fish link in the ocean supports some of the most productive ecosystems in the world and is an important source of highly nutritious food for upper trophic levels. Experimental studies indicate a weak sensitivity of primary production to CO_2_ [14] and no direct effects on copepod growth and hatching success at CO_2_ levels within the range expected by the end of this century [19], [20]. However, CO_2_ may indirectly affect zooplankton growth through its potential impact on the nutritional quality of phytoplankton, their major food source. To test this hypothesis, we independently manipulated CO_2_ concentration in both diatoms used as food algae and copepod cultures, and investigated dietary OA effects on copepod growth and reproduction. The experiment consisted of a two-by-two factorial design crossing two CO_2_ levels in food algae media and seawater used for copepod growth. The cryptophyte *Rhodomonas* sp. and diatom *Thalassiosira pseudonana* were used as food source and the copepod *Acartia tonsa* as consumer. We determined resulting FA composition of both alga and copepod as well as copepod development and reproduction. Our experiment showed that elevated CO_2_ affected biochemical composition of the diatom that constrained copepod growth performance.

## Methods

### CO_2_ manipulation and experimental design

The target values for experimental CO_2_ manipulation were 380 µatm for the low (L) and 740 µatm for the high (H) CO_2_ treatment. Phytoplankton (P) was grown at both L and H *p*CO_2_ concentrations and fed to copepod zooplankton (Z) grown in seawater at the same L and H target levels in a crossed design. It is important to note that biological activity, such as photosynthesis and respiration, alter the carbonate system. In addition, water exchange and combining treatments with low and high CO_2_, as was done in the copepod growth experiment, can result in deviations from the target CO_2_ levels. Nevertheless, *p*CO_2_ levels of L and H treatments were maintained close to target values and differences among treatments persisted throughout the experiment (Figure S1).

*Rhodomonas* sp. and *T. pseudonana* were cultured as food sources in artificial seawater at *p*CO_2_ of ∼495±100 SD (L) and ∼760±110 (H) for *Rhodomonas* sp. and ∼365±120 (L) and ∼915±270 (H) µatm for *T. pseudonana*, respectively. Juvenile copepods were fed with *Rhodomonas* to ensure optimal growth of the first developmental stages and *T. pseudonana* was used as food source after copepodite stage 1. The carbonate system of *T. pseudonana* cultures was manipulated by combined additions of sodium carbonate (Na_2_CO_3_) and hydrogen chloride (HCl) at constant alkalinity; the two CO_2_ treatments for *Rhodomonas* cultures were continuously aerated with CO_2_-enriched air. Algae were grown in laboratory batch cultures on a 18∶6 light∶dark cycle with replete nutrients. To investigate the response time of algae fatty acid composition alterations to changing *p*CO_2_, *T. pseudonana* was grown at high (∼1120 µatm) *p*CO_2_ for five days and then transferred to a low (∼380 µatm) *p*CO_2_ media. FA concentration was measured every five hours over a 30 h time period.

*Acartia tonsa* eggs were hatched in seawater under *p*CO_2_ conditions of ∼380 µatm. After the nauplii reached developmental stage 2, they were transferred into 2-L NALGENE bottles (1000 individuals L^−1^) filled with seawater (salinity 18.2) from a tank that was aerated continuously with appropriately CO_2_-enriched air of ∼495±100 (L) and ∼760±110 (H) µatm *p*CO_2_, respectively. Copepod zooplankton (Z) were fed with CO_2_ preconditioned phytoplankton (P) at about 1000 µg C L^−1^ in a factorial design with four treatment combinations: P_L_/Z_L_, P_L_/Z_H_, P_H_/Z_L_ and P_H_/Z_H_, each with three replicates. Water and food during the copepod growth experiment were replaced every other day. All replicates were randomly placed in a temperature-controlled culture room at 18°C and 14∶10h light∶dark cycle until the copepods reached adult stage. Over the course of the experiment, developmental stages were identified and at the end of the growth experiment egg production of females measured over 24 h and hatching success of eggs and nauplii morphological formation observed for two days. Species involved for this experiment were lab cultures and thus no specific permits were required for the sample collection.

Dissolved inorganic carbon (DIC) was measured after every water exchange and pH was recorded daily during the copepod growth experiment. For DIC the water was smoothly filtered via syringe and a 0.2 µm pre-filter and stored in 4 ml borosilicate flasks at 4°C. The sample flasks were closed with a plastic screw cap and a Teflon septum. DIC was determined photometrically with an auto-analyzer (QUAATRO, Bran & Lübbe) at a precision of ±20 µmol kg^−1^ [21], [22]. DIC and pH were used for seawater carbonate system calculations (Text S1). During the copepod growth experiment measured mean (±SD) pH values were 8.14±0.12 and 7.94±0.08, and for DIC 480±110 and 725±140 µatm CO_2_ in the L and H treatment, respectively (Figure S1). DIC values in the crossed treatments were 485±80 (P_H_/Z_L_) and 745±80 (P_L_/Z_H_) µatm CO_2_. Due to the fact that NBS based pH measurements are rather weak for reliable carbonate chemistry calculations, total alkalinity (TA) measurements (Text S1) were taken two times per week for crosscheck calculations. Values for *p*CO_2_ calculated from pH and DIC differed from *p*CO_2_ calculations using DIC and TA on average ∼110 and ∼210 µatm at the low and high CO_2_ treatment level, respectively, over the duration of the experiment. These uncertainties are probably higher than the real error since they were caused by outliers in TA and DIC measurements to which the carbonate system is relative insensitive when pH is involved in the calculations.

FA composition of *T. pseudonana* was analyzed from the stock culture during exponential growth phase and of copepod females at the end of the experiment. FAs were measured as fatty acid methyl esters (FAMEs) with a Thermo GC Ultra gas chromatograph equipped with a nonpolar column (RXI1-SIL-MS 0.32 µm, 30 m) using a flame ionization detector (FID). A complete method description is provided in Text S1.

### Statistical analysis

**Figure 1:**
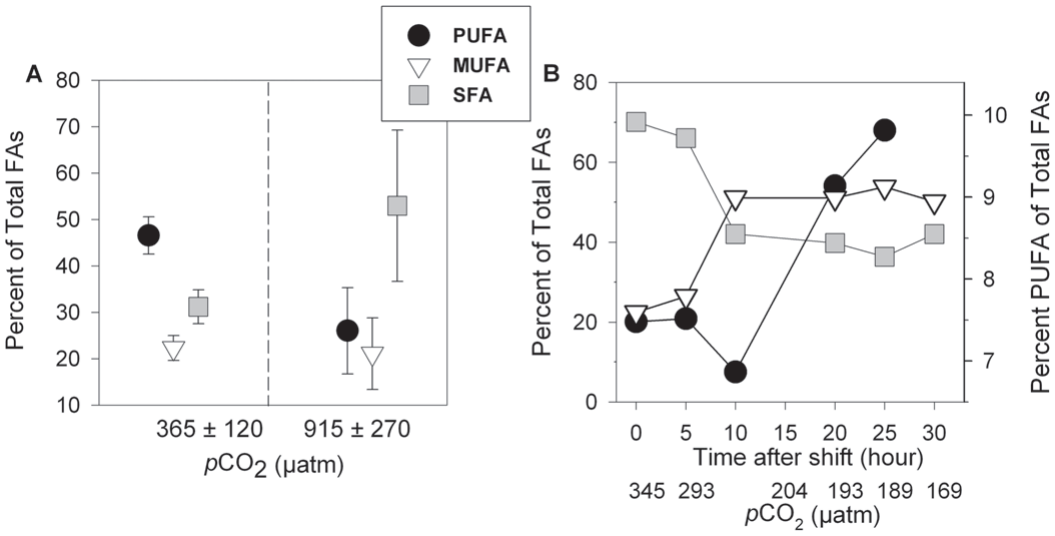
Fatty acid composition and concentration of *Thalassiorira pseudonana* cultured at different CO_2_ treatments. **A**) Percentage of polyunsaturated (PUFA), monounsaturated (MUFA), and saturated (SFA) fatty acids relative to total fatty acids during the exponential growth phase cultured at low (realized value of 365 µatm *p*CO_2_, n = 5) and high (realized value of 915 µatm *p*CO_2_, n = 3) CO_2_ treatments used as copepod food source. **B**) Change in the fatty acid composition in *T. pseudonana* after a shift from high to low *p*CO_2_ conditions (n = 1 per treatment level). Time 0 are measured values before the culture media shift. Error bars indicate standard errors.

Algal responses to experimental conditions were assessed using two-tailed *t*-tests. Differences in copepod FA classes and egg production between treatments were tested using analysis of variance (ANOVA). A Tukey HSD post hoc test was used to assess differences among treatments in egg production. Generalized linear models (GLM) were used to examine the effect of the seawater *p*CO_2_ used for algal and copepod cultures on the relative proportion of FA classes in copepods. Principal component analysis (PCA) was used to assess the difference in individual FA composition of the diet algae and copepods across the treatment combinations. For algal food, log-transformed FA concentration per cell and for copepods arcsine-square root transformed percentage of total FA was used since the proportion of FA classes varies between *T. pseudonana* and *A. tonsa* (see Figure 1). Each FA was standardized by subtracting its mean and dividing by its standard deviation, assembling the resulting standardized series into a 15-FA by 20-treatment combination data matrix. The PCA used a covariance matrix and Varimax rotation. This analysis identified FA that explained most to the observed variance. Statistical analyses were performed using Statistica and the R software environment 2.14.1 [23].

## Results and Discussion

Our experiment showed that CO_2_ concentration significantly changed FA concentration and composition in the diatom *T. pseudonana* used for copepod diet. The relative amount of PUFAs was significantly lower (*t* = 4.48, *p* = 0.004) and the amount of SFAs higher (*t* = −3.37, *p* = 0.015) at high *p*CO_2_ compared to the low *p*CO_2_ treatment (Figure 1A). Essential PUFA concentrations were significantly reduced at high *p*CO_2_ (Table S1), specifically DHA (22:6*n*-3; *t* = 2.81, *p* = 0.03) and the group ARA-EPA (20:4*n*-6, 20:5*n*-3; *t* = 6.63, *p*<0.001). A shift in FA composition at projected future CO_2_ levels is consistent with observations in the coccolithophorid *Emiliania huxleyi* [24] and with green algae and prymnesiophyte experiments conducted at extreme CO_2_ changes [25], [26], [27].

A separate experiment confirmed that the shift in FA occurred rapidly in response to changing *p*CO_2_ in the diatom *T. pseudonana*. When transferred from high to low CO_2_, FA composition was already significantly different from its initial composition after 15 h (Figure 1B) and FA components changed in the same direction as observed at constant high and low *p*CO_2_ treatments. Similarly, a rapid transition in FA composition can be expected when algae are transferred from low to high *p*CO_2_, which was, however, not tested in our experiment. Though, a rapid reversible FA response to changing *p*CO_2_ concentration has been reported in green algae [26]. The higher unsaturation levels of FAs in algae cells cultured at low *p*CO_2_ compared to cells at high *p*CO_2_ has been suggested to be partially a consequence of repressed FA synthesis, which promotes the desaturation of pre-existing SFAs [26]. Recently it has been proposed that pH might act as a regulation signal for the formation of cell membranes, which are mainly composed of fatty acids, by controlling the production of its synthesizing enzymes [28]. A high environmental *p*CO_2_ (low pH) can decrease the internal cell-pH [29]. Therefore the increased amount of SFAs could be a mechanism to control the internal cell-pH, as a membrane built of short-chain FAs is less fluid and permeable to CO_2_. However, the cellular processes involved in FA synthesis under changing pH or *p*CO_2_ levels are not fully understood.

**Figure 2:**
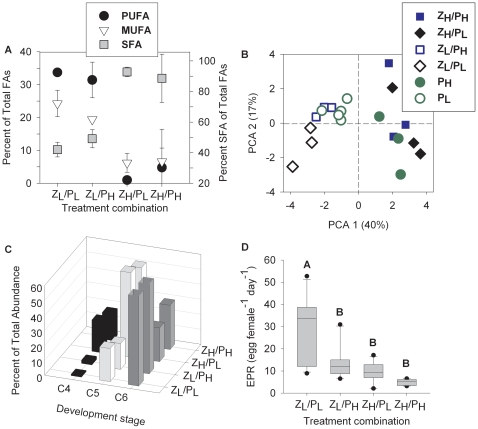
Fatty acid composition, somatic growth and reproduction of *Acartia tonsa* across CO_2_ treatment combinations. **A**) Percentage of polyunsaturated (PUFA), monounsaturated (MUFA), and saturated (SFA) fatty acids relative to total fatty acids in female copepods. **B**) Principal component analysis (PCA) of fatty acid composition for the dietary algae *Thalassiorira pseudonana* and *A. tonsa* of the different treatment combinations. PCA scores 1 explained 40% of the variability (see *x*-axis of c) and was highly negatively correlated with 22:6*n*-3 (r^2^ = 0.73), 20:4*n*-6+20:5*n*-3 (r^2^ = 0.85), 18:3*n*-6 (r^2^ = 0.73) and 16:1 (r^2^ = 0.79), and positively with 22:1*n*-9 (r^2^ = 0.25) and 18:1*n*-9*t* (r^2^ = 0.57). PCA score 2 explained 17% of the overall variability (see *y*-axis of c) and was strongest positively correlated with 24:0 (r^2^ = 0.84). Loadings of the PC scores are shown in Figure S2). **C**) Stage distribution of *A. tonsa* individuals at day 10. C4, C5, C6 = copepodite stage 4, 5, and adult, respectively. **D**) Egg production rate (EPR) of incubated females (n = 12 per treatment level). EPR was significantly different between treatments (F_(3, 44)_ = 18.02, *p*<0.001). Different letters above bars represent significant differences from a Tukey HSD test. The bars represent the 25^th^, 50^th^ and 75^th^ percentiles, whiskers stand for the 10^th^ and the 90^th^ percentiles and black points show outliers. Legend refers to treatment combinations of copepod zooplankton (Z) and phytoplankton food source (P) at low (L) and high (H) *p*CO_2_.

Similar to FA modification in algal food, FA concentration and composition of adult copepods varied significantly between CO_2_ treatments. The mean ±SD total amount of FAs in *A. tonsa* was significantly different across treatments (*F*_(3, 8)_ = 5.15, *p* = 0.028) and higher when raised and fed with algae cultured at low *p*CO_2_, with 8.9±5.6 ng ind.^−1^ compared to 0.8±0.2 ng ind.^−1^ when both copepods and algal diet were cultured at high *p*CO_2_ and to 2.3±0.5 ng ind.^−1^ in the crossed treatment combinations (Table S1). Copepods raised and fed with algae at low *p*CO_2_ contained high proportions of PUFAs relative to total FAs that are in the same range with reports in marine calanoids [30]. The PUFA fraction in copepods decreased from more than 30% at low *p*CO_2_ to less than 5% at high *p*CO_2_ (*F*_(3, 8)_ = 54.51, *p*<0.001) (Figure 2A). The long-chain highly unsaturated FAs DHA and ARA-EPA, which are important components for growth and reproduction of consumers [31], decreased from 15% in copepods raised at low *p*CO_2_ below detection limit in those at high *p*CO_2_ (Table S1). Similarly, the proportion of MUFAs (monounsaturated fatty acids) varied significantly across treatments (*F*_(3, 8)_ = 8.2, *p* = 0.008) and decreased from around 20% at low *p*CO_2_ to less than 10% at high *p*CO_2_. On the other hand, the relative amount of SFAs tripled in copepods at high *p*CO_2_ (Figure 2A) and FA compositions were different between treatments (*F*_(3, 8)_ = 26.22, *p* = <0.001).

Contrary to our expectation, FA composition in copepods differed between individuals raised at low and high seawater *p*CO_2_, irrespective of the CO_2_ level of their algal diet (Figure 2A). Because consumers are unable to synthesize PUFAs we expected that copepod FA composition in the crossed *p*CO_2_ treatments of copepod culture and food algae (P_L_/Z_H_, P_H_/Z_L_) would reflect changes in FA of their diet. Principal component analysis (PCA) of individual FAs in diet algae and copepods also showed distinctive clustering of the copepod groups raised at low and high *p*CO_2_ treatments, irrespective of the CO_2_ conditions of their diet algal culture (Figure 2B), which was mainly explained by PUFAs and SFAs (Figure S2). A GLM model supported that CO_2_ concentration of the seawater used to raise copepods significantly negatively affected the relative proportion of PUFAs (*p*<0.001) and positively affected the proportion of SFAs in copepods (*p*<0.001), which was consistent across combinations and not dependent on the *p*CO_2_ level of the algal culture.

These findings suggests that the FA composition of algae changed rapidly when transferred from low *p*CO_2_ culture media to high *p*CO_2_ seawater used to raise copepods and *vice versa*. Since consumers are unable to synthesize PUFAs [30] and previous experiments showed that copepod growth is rather insensitive to CO_2_ levels within OA predictions [19], [20], direct CO_2_ effects on copepod FA synthesis seem unlikely. In our experiment, water and food was exchanged every second day and algae were in their exponential growth. Thus, we rather expect that high turnover rates and the ability of *T. pseudonana* to rapidly change the FA composition in a variable *p*CO_2_ environment (Figure 1B) are responsible for an adjustment in FA composition in the crossed treatments within the first day. Rapid modification in algae FA and the fact that *A. tonsa* has no lipid reserves [32] likely explains the absence of the influence from the algae culture media *p*CO_2_ on copepod FA composition within both crossed treatment combinations.

The CO_2_-dependent dietary shift in FAs had a significant effect on *A. tonsa* growth and development. Copepods of the same age (10 d) showed a delay in stage development of 1 to 2 days at high *p*CO_2_ (Figure 2C). Egg production decreased from a median of 34 eggs female^−1^ d^−1^ at low water and food *p*CO_2_ to less than 12 eggs female^−1^ d^−1^ in all other treatments, with the lowest production (5 eggs female^−1^ d^−1^) at high water and food *p*CO_2_ (Figure 2D). The egg production rate was significantly related to the ratio of PUFA:SFA and the content of DHA and ARA-EPA within the female copepods (Table 1), consistent with other observations in zooplankton [33]. Copepod egg production raised at low *p*CO_2_ and fed with algae grown at high *p*CO_2_ produced significantly less eggs compared to copepods in the low *p*CO_2_ treatment combination (Figure 2D). This significant decline is most likely a result of the overall lower copepod FA quantity when fed with algae cultured at high CO_2_ compared to food at low CO_2_ (Table S1). Given that adult *A. tonsa* females invest the majority of their lipids into reproduction [34], the significant decrease of essential PUFAs due to low quality food algae is most likely the reason for the considerable decline in egg production observed in the high *p*CO_2_ treatment combinations (Figure 2).

**Table 1:** Regression statistics of *Acartia tonsa* egg production as a linear function of fatty acid composition.

Fatty acid	Slope	*Y*-intercept	r^2^	*p-value*
PUFA (%)	0.03	1.9	0.52	0.013
MUFA (%)	0.07	1.6	0.73	**<0.001**
SFA (%)	−0.02	3.8	0.60	**0.005**
PUFA:SFA	1.3	1.9	0.59	**0.006**
ARA-EPA (ng cop^−1^)	0.68	2.06	0.67	**0.002**
DHA (ng cop^−1^)	1.23	1.92	0.77	**<0.001**

Bonferroni-corrected significance levels for multiple fatty acid comparisons were α = 0.008 (0.05/6). Significant correlations are highlighted in bold; n = 11. PUFA = polyunsaturated fatty acid; MUFA = monounsaturated fatty acid; SFA = saturated fatty acid; ARA-EPA = 20:5*n*3; DHA = docosahexaenoic acid (22:6*n*3).

Some studies showed that the disruption of diatom cells induced by feeding triggered the transformation of unsaturated FAs into aldehydes causing adverse effects on copepod development, egg production and hatching success [35]. Even though several diatom species are known to possess these deleterious effects, *T. pseudonana* has recently been reported not to produce aldehydes [36]. We also could not find any malformations of the hatched nauplii by microscopic observations (data not shown), suggesting that negative aldehyde effects were not present.

Our study suggests that OA can have important consequences for consumer growth and production by affecting the nutritional quality of primary producers that translates to higher trophic levels. These results are consistent with experiments on freshwater cladocerans, fed with algae from an acidic lake [37], suggesting that our results are not restricted to monospecific laboratory cultures and may be expected at community level. However, future experimental manipulations are required to clarify the widespread response of phytoplankton biochemical composition to ocean acidification at relevant *p*CO_2_ levels in other taxonomic groups and natural communities. It can be expected that trophic upgrading and differential algae sensitivity to *p*CO_2_ at the community and ecosystem level may compensate for low food quality observed at the single species level. Moreover, the tolerance to *p*CO_2_ and pH might be lower for monocultures compared to natural populations, which have high ecophysiological variability [38] and genetic diversity, important for adaption to various environmental factors [39]. Nonetheless, shifts in FA composition as a response to changing CO_2_ have been documented in other phytoplankton species [26], [40], and FA-responses in phytoplankton as observed here might be important during bloom periods if CO_2_ sensitive organisms dominate.

The effect of OA on nutritional quality in the diatom-copepod food chain relationship observed in our study may have far reaching consequences for food webs since FAs originating in phytoplankton are sequentially incorporated into the total lipid fraction of zooplankton and triacylglycerol of larval fish [41]. Given that fish is a critical natural resource [42], acidification-driven food quality deterioration may impair fish production by changing the biochemical composition of food algae and its transfer to higher trophic levels [43], [44]. While it is difficult to extrapolate from monocultures to community level, these results point to the likelihood that OA consequences go beyond direct physiological impacts and that indirect effects through trophic interactions need to be considered.

## Supporting Information

Figure S1 Carbonate system over the course of the copepod growth experiment.
(DOCX)

Figure S2 Loadings for Principal Component Analysis (PCA) of fatty acids for *Thalassiosira pseudonana* and *Acartia tonsa*.
(DOCX)

Text S1 Full material and methods description.
(DOCX)

Table S1 Amount of fatty acids of the food algae *Thalassiosira pseudonana* and the copepod consumer *Acartia tonsa* at different CO_2_ treatment combinations.
(DOCX)
