# Supplementary material for: Ocean Acidification-Induced Food Quality Deterioration Constrains Trophic Transfer
Source: PLoS One. 2012 Apr 11;7(4):e34737. doi: 10.1371/journal.pone.0034737 (PMC3324536; doi:10.1371/journal.pone.0034737)
Supplement: Figure S1 — Carbonate system over the course of the copepod growth experiment. (DOCX) [file pone.0034737.s001.docx]

**
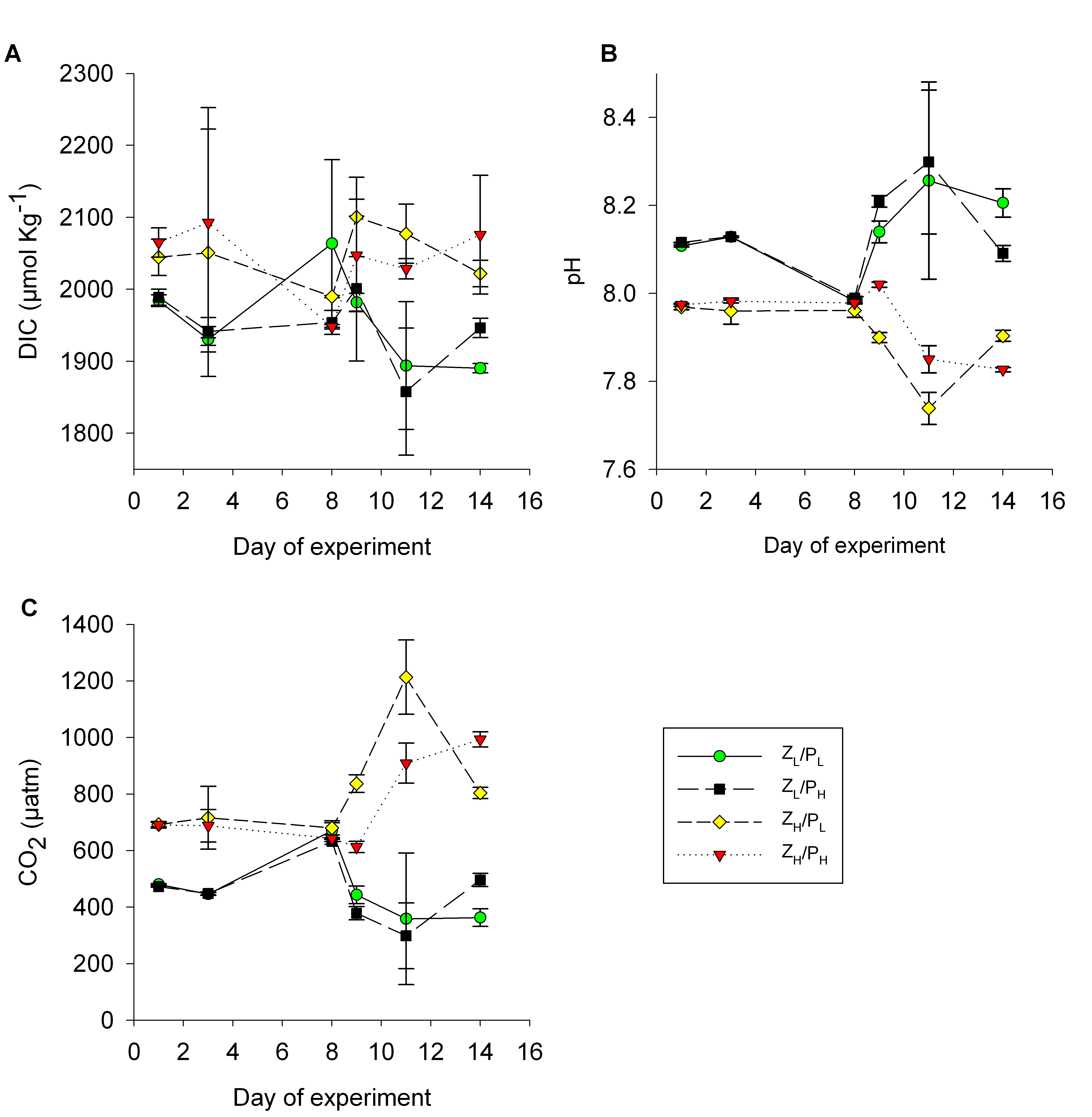
**

**Figure S1.** Carbonate system over the course of the copepod growth experiment.

**A)** Dissolved inorganic carbon (DIC) within CO_2_ treatments during experimental growth phase of the copepods with a standard deviation of 47-91 µmol kg^-1^ within replicates of each treatment combination. On the first and the last day, all treatments were well separated with some overlap on days 8 and 9. In the high CO_2_ treatment (Z_H_/P_H_), incubation bottle 740-4 displayed maximum deviation of DIC with 130-210 µmol kg^-1^ relative to the other treatment replicates. **B)** Mean pH values during experimental growth phase of the copepods were well separated between the two main CO_2_ seawater treatments 8.14 and 7.94 in the Z_H_ and Z_L_ treatment, respectively, with standard deviations ranging from 0.08 to 0.12. **C)** Calculated mean CO_2_ values in Z_L_ and Z_H_ were 460 and 755 µatm, respectively, with a standard deviation of 3-57 µatm between replicates during the first feeding period with *Rhodomona salinas*. From day 8 on, after the food algae was changed from *R. salinas* to *Thalassiosira* ***pseudonana***, the standard deviations of the mean CO_2_ values in all treatment combinations were higher, ranging from 20 to 232 µatm. The CO_2_ concentrations of the mixed treatment combinations Z_L_/P_H_ and Z_H_/P_L_ were closer to the CO_2_ target levels with 454 and 823 µatm, respectively. Error bars indicate standard deviations.
