# Supplementary material for: Ocean Acidification-Induced Food Quality Deterioration Constrains Trophic Transfer
Source: PLoS One. 2012 Apr 11;7(4):e34737. doi: 10.1371/journal.pone.0034737 (PMC3324536; doi:10.1371/journal.pone.0034737)
Supplement: Figure S2 — Loadings for Principal Component Analysis (PCA) of fatty acids for Thalassiosira pseudonana and Acartia tonsa. (DOCX) [file pone.0034737.s002.docx]

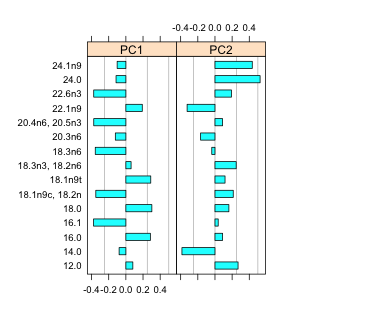


**Figure S2.** Loadings for Principal Component Analysis (PCA) of fatty acids for *Thalassiosira* *pseudonana* and *Acartia tonsa*.

FAs 22.6n3, 20.4n6.20.5n3, 18.3n6, 18.3n3-18.2n6, and 16.1 contributed most to Axis 1, and 24.0 and 14.0 to Axis 2. PCA is shown in Figure 2b.
