# Supplementary material for: Ocean Acidification-Induced Food Quality Deterioration Constrains Trophic Transfer
Source: PLoS One. 2012 Apr 11;7(4):e34737. doi: 10.1371/journal.pone.0034737 (PMC3324536; doi:10.1371/journal.pone.0034737)
Supplement: Text S1 — Full material and methods description. (DOCX) [file pone.0034737.s003.docx]

**Supporting Information:**

**Text S1.** Full material and methods description

**Manipulation of the carbonate chemistry**

The target values for experimental CO_2_ manipulation were 380 µatm in the low (L) and 740 µatm *p*CO_2_ in the high (H) CO_2_ treatment. To adjust seawater and the culture media to the different *p*CO_2_ levels, the carbonate system was manipulated using two methods, depending on the sensitivity of the organisms to *p*CO_2_ manipulation [[1](#_ENREF_1)]: 1) For *Thalassiosira pseudonana* cultures, carbonate chemistry was adjusted by combined additions of sodium carbonate (Na_2_CO_3_) and hydrogen chloride (HCl), manipulating dissolved inorganic carbon (DIC) while keeping total alkalinity (TA) constant because diatoms can be negatively affected by aerating. Artificial seawater was used as culture media (recipe after [[2](#_ENREF_2)]). 2) For copepods and *Rhodomonas* *salina* used as nauplii diet, *p*CO_2_ was manipulated by aeration of seawater and culture media at a target *p*CO_2_ concentration. This was achieved by bubbling culture vessels with a mixture of CO_2_ and air with the respective target values. The difference between acid addition and bubbling is a small difference in HCO_3_ concentration, while pH and concentrations of CO_2_ and CO_3_ are the same [[1](#_ENREF_1)].

### CO_2_ enrichment of seawater stock solution for the copepod growth experiment

Baltic seawater from Kiel Fjord was filtered with a 0.2 µm SartobranP sterile MidiCap membrane filter and stored in two 300 L tanks, bubbled continuously with aeration stones using ambient air (*p*CO_2_ = 380 ppm) and CO_2_-enriched air (*p*CO_2_ = 740 ppm). All CO_2_ enrichments were undertaken with a CO_2_-gas mixing system (Linde GMZ 750 IRA infrarot absorption sensor). The seawater tanks (salinity 18.2) were closed with a rounded plexiglass lid (Adolf Richter GmbH) and stored in darkness in a temperature-controlled culture room at 18°C. Water pH was monitored daily using a WTW 340i pH-analyzer connected to a SENTIX-81 electrode. The electrode was calibrated with standard WTW buffer 4 and 7 before each measurement set.

### Copepod growth experimental set-up

The copepod *Acartia tonsa* was used as consumer. *A. tonsa* eggs obtained from the Alfred Wegener Institute for Polar and Marine Research (Helgoland) were initially incubated for 3 days within a 200 L tank filled with 0.2 µm filtered seawater from Kiel Fjord (salinity 18.2, temperature 18 °C). After the hatched nauplii reached the second developmental stage, individuals were transferred from the stock solution to 2-L NALGENE bottles filled with CO_2_ pre-treated water to reach an approximate density of 1000 individuals L^-1^ in each treatment and replicate (12 bottles total). In a full factorial design, nauplii were grown under two different levels of CO_2_ aerated seawater (low and high; see above) and fed with algae at a concentration of 1000 µg C L^-1^ cultured under low and high CO_2_ levels (see below). Nauplii were fed with CO_2_ aerated stock *R.* *salina* to ensure optimal growth and development of the first developmental stages. After the copepods reached copepodide stage 1, *T. pseudonana* was used as food source. The CO_2_ manipulation resulted in a mean (±SD) *p*CO_2_ concentration in the L and H treatment of 533 (±28) and 674 (±57) µatm during the *R.* *salina* food addition and 388 (±233) (L) and 838 (±70) (H) µatm during the *T. pseudonana* addition. Four treatment combinations for copepod zooplankton (Z) and phytoplankton diet (P) were used: Z_L_/P_L_, Z_L_/P_H_, Z_H_/P_L_ and Z_H_/P_H_ (each in triplicate). All replicates were randomly placed in a temperature-controlled culture room for 16 days at 18 °C and 14 h:10 h light:dark cycle until the copepods reached the adult stage.

After the copepods reached copepodite stage 1 the total water of all incubation bottles was exchanged. Therefore the content of each flask was slowly run through a 41 µm mesh to separate the copepods from *R.* *salina*. The copepods were then smoothly rinsed and transferred into clean incubation bottles, refilled with CO_2_ aerated seawater. For the following growth phase (copepodite stage 2 to adult), *T. pseudonana* stock culture was added as diet following the CO_2_ treatment combinations. Microscopic inspection of water samples taken directly after the refilling process from each incubation bottle ensured no further contamination with *R.* *salina*.

During the course of the copepod growth experiment, 80–90 % incubation water was exchanged with CO_2_-enriched seawater from the stock solution tanks every second day to ensure a constant CO_2_ environment. Water was removed by reverse filtration using an 80 cm silicone tube with a net ending of 41 µm mesh.

### Carbonate chemistry sampling and analysis

Water pH was monitored daily over the course of the copepod growth experiment. Water samples for carbonate chemistry analysis were taken every second day for DIC and two times per week for TA, respectively directly after water exchange. For DIC, the water was smoothly filtered via syringe and a 0.2 µm pre-filter and stored in 2 ml brown flasks at 4 °C. The sample flasks were closed with a plastic screw cap and a Teflon septum in between to minimize outgassing. DIC was determined photometrically with an auto-analyzer (QUAATRO, Bran & Lübbe) at a precision of ±20 μmol kg^−1^ [[3](#_ENREF_3)], [[4](#_ENREF_4)]. Samples for TA measurements were filtered, poisioned with 1 ml of a HgCl_2_ solution (35 g L^-1^) and stored in 300 ml borosilicate flasks. TA was calculated from linear Gran plots [[5](#_ENREF_5)] after duplicate potentiometric titration [[6](#_ENREF_6)].

The values of DIC and pH (NBS scale), combined with temperature and salinity, were used to calculate carbonate system parameters in the seawater. CO_2_ calculations were conducted with an excel macro of the CO2SYS.EXE program [[7](#_ENREF_7)] using the constants after [[8](#_ENREF_8)] as refitted after [[9](#_ENREF_9)] and [[10](#_ENREF_10)]. pH measurements were checked with alkalinity measurements two times per week by calculating pH from measurements of TA and DIC using the software CO2sys (see above). Calculated pH values agreed with pH measurements with a maximum deviation of ±110 and ±210 µatm for the low and high CO_2_ treatment level, respectively.

### Egg production and hatching experiments

After copepods reached adult stage, *A. tonsa* females were sorted from each incubation bottle and transferred into 500 ml chambers filled with CO_2_-treated seawater and *T. pseudonana* (1000 µg C L^-1^) and closed leaving no headspace. To separate the copepods from produced eggs, a mesh of 250 µm separated the chambers. For each experimental replicate, 4 egg chambers each with 5 females were set up (total 48 egg chambers).

After 24h incubation time all living female copepods were separated from the egg chambers and stored under -80°C for fatty acid analysis. The eggs and hatched nauplii inside the egg chambers were concentrated and transferred into 20 ml airtight hatching chambers for another incubation of 48h, followed by formalin preservation. The content of the hatching chambers was analyzed and categorized into nauplii, hatched and empty eggs; nauplii were further examined for visible mutation or developmental malfunctions. All procedures of the egg production and hatching experiment took place within a temperature-controlled culture room at constant temperature conditions (18° C). Copepod sorting and analysis took place with a Leica MS 5 microscope (10–40x).

### Preparation of algae stock cultures

The cryptophyte *R. salina* and the diatom *T. pseudonana* were used as food sources for *A. tonsa*. *R. salina* was inoculated and grown in two flasks with a modified f/2 media (salinity 18.2) and bubbled continuously with CO_2_ air of 380 ppm (low) and 740 ppm (high), respectively under constant light. *T. pseudonana* was grown in a set of laboratory batch cultures with artificial seawater (ASW) as culture media [[2](#_ENREF_2)] at a salinity of 18 and constant alkalinity of 2041 μmol kg^-1^ to simulate the parameters measured in the copepod media. TA and DIC were measured in the *T. pseudonana* cultures before its use for copepod feeding. The carbonate system was calculated in the same way as was done in the copepods media, resulting in a mean (±SD) *p*CO_2_ of 366±120 and 915±270 µatm in the L and H treatment, respectively. The relatively high *p*CO_2_ variability within each treatment is expected to be a consequence of DIC losses and slight changes in alkalinity in the culture media due to cell growth. Incubations were conducted at a photon flux density of 150 μmol photons m^-2^ s^-1^ induced by six fluorescent tubes [T5, types 5 JBL Solar Tropic (4,000 K), 1 JBL Solar Natur (9000 K)] on a 18:6h light:dark cycle, and a temperature of 18°C. Nitrate, silicate, and phosphate were added at final concentrations of approximately 64, 64, and 4 μmol kg^-1^, respectively; trace metals and vitamins were added at f/2 medium concentrations [[11](#_ENREF_11)].

**Algae CO_2_ shift experiment**

To investigate the response through time of the algae fatty acid composition to changing *p*CO_2_, *T. pseudonana* was grown in a batch culture at 1120 µatm *p*CO_2_ under the same light and temperature conditions as the copepod experiment. Nutrients were set at the same concentrations used in the algae cultures for copepod feeding. After 5 days the culture was transferred to a medium with 380 µatm *p*CO_2_. The further decline in *p*CO_2_ after the transfer to low *p*CO_2_ was a consequence of the carbon dioxide uptake by the algae and was accentuated by sporadic vacuum pumping of the culture bottle overhead space; the *p*CO_2_ varied between 345 µatm at the beginning to 169 µatm at the end of the experiment. TA and DIC were measured and the carbonate system calculated as was done in the copepods media. Samples for fatty acids were taken every 5 hours over a 30h period, with a pause at 15 hour during the dark phase of the culture. Cells were harvested during the exponential growth phase of the culture. Algal density was determined using a Coulter Counter (Z2 Coulter® Particle Count and Size Analyzer, Beckman Coulter™).

### Fatty acid analysis

Fatty acids of the copepods and algae food culture were measured as fatty acid methyl esters (FAMEs) at the end of the egg production experiment. 250 or 500 ml of water, depending on the concentration of phytoplankton, were filtered on precombusted 0.2 µm GF/F filters and stored at -80°C until analysis. 45-50 adult copepods from each incubation bottle were sampled into 2 ml scintivials and stored at -80°C until analysis. Lipids were extracted over night from the filters using 3ml of a solvent mixture (dichloromethane:methanol:chloroform in 1:1:1 volume ratios). As internal standard, a five component FAME Mix (company Restek, Bad Homburg, Germany; c= 18.09 ng component^-1^µl^-1^) was added, and a C23 FA standard (c= 25.1 ng µl^-1^) was used as an esterification efficiency control (usually 80-85 %). Water-soluble fractions were removed by washing with 2.25 ml of KCl solution (c= 1 mol L^-1^), and the remainder dried by addition of NaSO_4_. The solvent was evaporated to dryness in a rotaryfilm evaporator (100-150 mbar), redissolved in Chloroform and transferred into a glass cocoon. Again, the solvent was evaporated to dryness (10-30mbar), and esterification was performed overnight using 200µl 1% H_2_SO_4_ (in CH3OH) and 100µl toluene at 50°C. Phases were split using 300µl 5% sodium chloride solution, and FAMEs were separated using n-Hexane, transferred into a new cocoon, evaporated, and 100µl (final volume) added. All solvents used were gas chromatography (GC) grade. FAMEs were analyzed by a Thermo GC Ultra gas chromatograph equipped with a nonpolar column (RXI1-SIL-MS 0.32µm, 30m, company Restek) using a FID. The column oven was initially set to 100°C, and heated to 220°C at 2°Cmin^-1^. The carrier gas was helium at a constant flow of 2ml min^-1^. The flame ionization detector was set to 280°C, with a gas flow of 350, 35 and 30 ml min^-1^ of synthetic air, hydrogen, and helium respectively. Injected were 1-µl aliquots of the samples. The system was calibrated with a 37-component FAME-mix (company Supelco, Germany) and chromatograms were analyzed using Chrom-Card Trace-Focus GC software.

**References**

1. Riebesell U, Fabry V, Hansson L, Gattuso J (2010) Ocean Acidification Guide: Guide to best practices for ocean acidification research and data reporting: Report: EUR 24328 EN.

2. Kester DR, Duedall IW, Connors DN, Pytkowics RM (1967) Preparation of artificial seawater. Limnology and Oceanography.

3. Stoll MHC, Bakker K, Nobbe GH, Haese RR (2001) Continuous-flow analysis of dissolved inorganic carbon content in seawater. Analytical Chemistry 73: 4111–4116.

4. Dickson A, Afghan J, Anderson G (2003) Reference materials for oceanic CO_2_ analysis: a method for the certification of total alkalinity. Marine Chemistry 80: 185-197.

5. Gran G (1952) Determination of the equivalence point in potentiometric titrations of seawater with hydrochloric acid. Oceanologica Acta 5: 209–218.

6. Bradshaw A, Brewer P, Shaffer D, Williams R (1981) Measurements of total carbon dioxide and alkalinity by potentiometrc titration in the GEOSECS program. Earth and Planetary Science Letters 55: 99–115.

7. Lewis E, Wallace DWR (1998) Program developed for CO_2_ system calculations. ORNL/CDIAC-105. Carbon Dioxide Information Analysis Center, Oak Ridge National Laboratory, U.S. Department of Energy, Oak Ridge, Tenn., U.S.A.

8. Mehrbach C, Culberson C, Hawley J, Pytkowicz R (1973) Measurement of the apparent dissociation constants of carbonic acid in seawater at atmospheric pressure. Limnology and Oceanography 18: 897-907.

9. Dickson AG, Millero FJ (1987) A comparison of the equilibrium constants for the dissociation of carbonic acid in seawater media. Deep Sea Research Part A Oceanographic Research Papers 34: 1733-1743.

10. Millero F, Graham T, Huang F, Bustos-Serrano H, Pierrot D (2006) Dissociation constants of carbonic acid in seawater as a function of salinity and temperature. Marine Chemistry 100: 80-94.

11. Guillard R, Ryther J (1962) Studies of marine planktonic diatoms. Canadian Journal of Microbiology 8: 229–239.
