# Supplementary material for: Ocean Acidification-Induced Food Quality Deterioration Constrains Trophic Transfer
Source: PLoS One. 2012 Apr 11;7(4):e34737. doi: 10.1371/journal.pone.0034737 (PMC3324536; doi:10.1371/journal.pone.0034737)
Supplement: Table S1 — Amount of fatty acids of the food algae Thalassiosira pseudonana and the copepod consumer Acartia tonsa at different CO2 treatment combinations. (DOCX) [file pone.0034737.s004.docx]

Table S1. Amount of fatty acids in *Thalassiosira* *pseudonana* (Phytoplankton, P) and in copepod zooplankton (*Acartia tonsa*, Z) cultured in seawater at low (L) and high (H) CO_2_ concentration in a full factorial design.


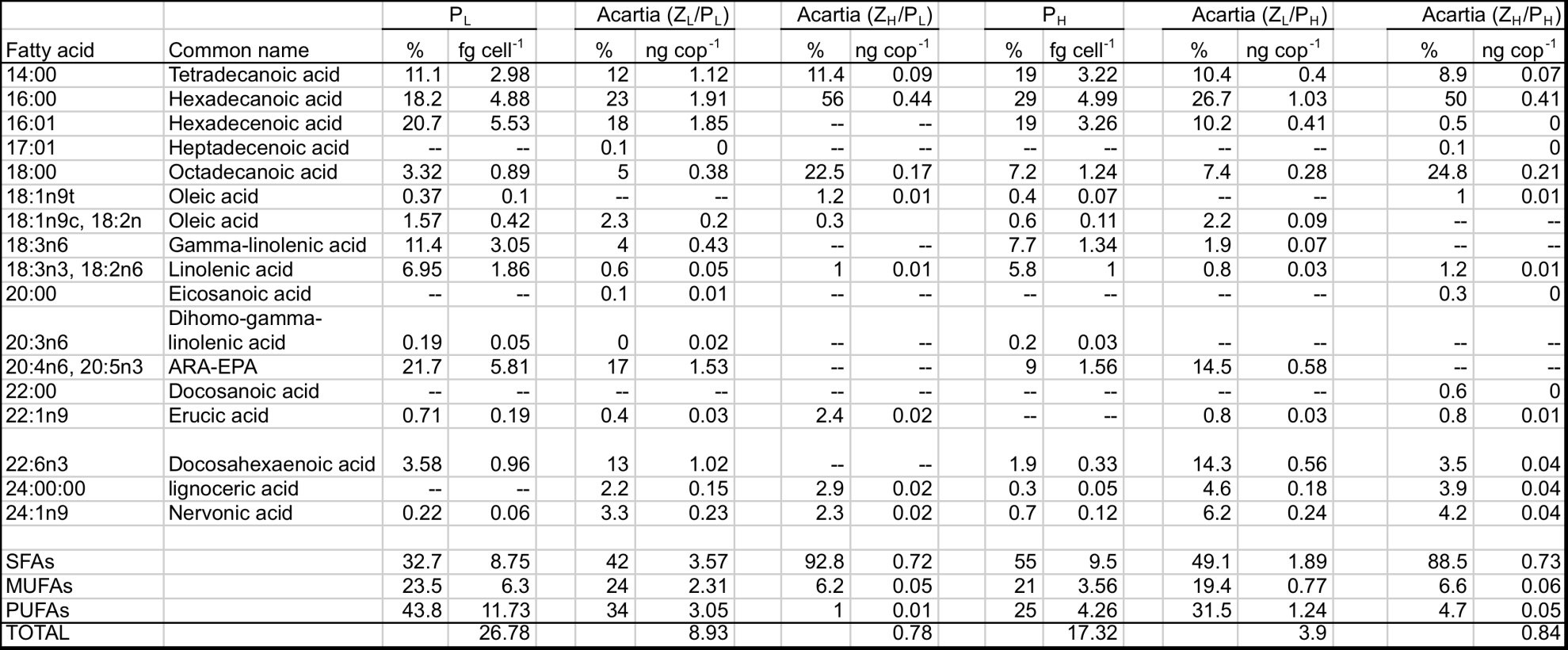


Realized *p*CO_2_ values were 497 µatm ± 98 sd for the low (Z_L_/P_L_) and 761 µatm ± 113 for the high (Z_H_/P_H_) treatment. Shown are percentage of total fatty acids and concentration per cell (fg) and per copepod (ng), respectively
